# Endotoxins from a Pharmacopoeial Point of View

**DOI:** 10.3390/toxins10080331

**Published:** 2018-08-16

**Authors:** Elvira Franco, Verónica Garcia-Recio, Pilar Jiménez, Manuel Garrosa, Tomás Girbés, Manuel Cordoba-Diaz, Damián Cordoba-Diaz

**Affiliations:** 1 Pharmaceutics and Food Technology, Complutense University of Madrid, 28040 Madrid, Spain; elvirafg@farm.ucm.es (E.F.); vgrecio@ucm.es (V.G.-R.); 2 University Institute of Industrial Pharmacy (IUFI), Complutense University of Madrid, 28040 Madrid, Spain; 3 Area of Nutrition and Food Sciences, University of Valladolid, 47005 Valladolid, Spain; pilarj@bio.uva.es (P.J.); girbes@bio.uva.es (T.G.); 4 Area of Histology, Faculty of Medicine and INCYL, University of Valladolid, 47005 Valladolid, Spain; garrosa@med.uva.es

**Keywords:** endotoxins, pyrogens, parenteral drug products, pharmacopoeial test, harmonization

## Abstract

A pyrogen is a substance that causes fever after intravenous administration or inhalation. Gram negative endotoxins are the most important pyrogens to pharmaceutical laboratories. In the International, United States, Japanese and European Pharmacopoeias, there are two official methods to evaluate pyrogenicity—that is, the bacterial endotoxin test, and the pyrogen test. The main objective of this review is to compare the monographs of each test among the different Pharmacopeias, to detect similarities and differences. The former can be considered fully harmonized, and only non-significant differences were detected. The latter, which is the only available assay for some products and formulations to demonstrate apyrogenicity, shows large differences, which should be considered.

## 1. Introduction

**Figure 1:**
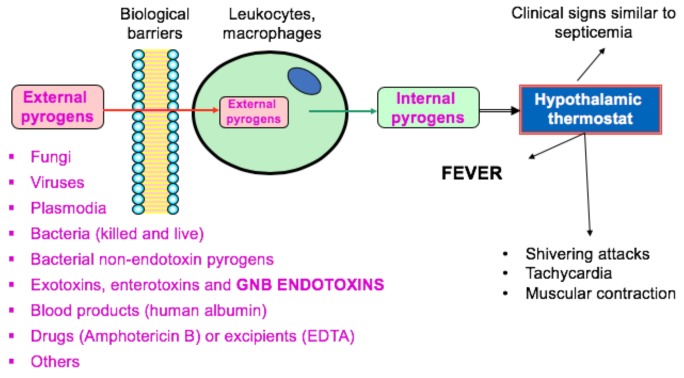
Most significant pyrogens for pharmaceutical manufacturers and sequential events of fever. GNB = Gram-negative bacteria.

Pyrogens are defined as substances that cause exacerbate febrile reactions when sufficient amounts gain access to the circulatory system after parenteral administration or inhalation. Although “pyrogen” is a general term to define fever-producing agents (Figure 1), it is frequently used to specifically describe Gram-negative bacteria (GNB) endotoxins in the pharmaceutical industry. Other pyrogenic substances can occur, although the risks are lower [1].

GNB endotoxin is a high molecular weight complex that contains lipopolysaccharide (LPS), protein, and phospholipid originating from the outer membrane of Gram-negative bacteria. Most pharmacopoeial endotoxin reference standards should be more correctly described as purified LPS since its chemical nature after purification is a lipid component called Lipid A, covalently bound to a polysaccharide composed of two parts, the core and a variable O-specific side chain, responsible for the specific immune reaction evoked in the host.

Depyrogenation is one of the most important challenges for pharmaceutical manufactures of parenteral drugs, since fever in a patient depends on the total amount of pyrogen delivered to that patient. Dry heat at temperatures above 180 °C is the method of choice for heat-resistant products, since GNB endotoxins are thermostable in the presence of moist heat and are not significantly destroyed by conventional autoclaving processes [2]. Moreover, another interesting property of GNB endotoxin is its tendency to aggregate into vesicles due to the attraction between hydrophobic groups of the LPS. These vesicles are large enough to be removed by reverse-osmosis processes or size exclusion chromatography. It should be considered, that in an aqueous environment the endotoxin aggregation state depends on its surrounding environment, i.e., divalent cations such as calcium or magnesium forms larger, more stable and lower soluble endotoxin aggregates. This property can be of particular interest in depyrogenation by ultrafiltration processes. Utilizing the electrostatic properties of GNB endotoxin can offer another interesting alternative for depyrogenation. It has been described that endotoxins are positively charged at pH levels above 5, and negatively charged at pH levels under 2. This property is very useful since it accounts for the attraction that GNB endotoxins have for stationary phases in chromatographic isolation [3].

The origins of the rabbit pyrogen test data back to studies by Seibert, who developed a mammalian test between 1923 and 1925, in response to concerns from surgeons about fever [4]. In 1943, Welch and coworkers published two reference papers outlining the results of a collaborative study performed by the U.S. Food & Drug Administration (FDA), the National Institutes of Health (NIH), and several pharmaceutical companies [5,6], resulting in the first rabbit pyrogen test (RPT) to be included in the United States Pharmacopoeia (USP). The current USP monograph, based on the evaluation of the rectal temperature of rabbits before and after an intravenous injection of a test solution into the ear, is not substantially different from the original one.

The bacterial endotoxin test (BET)—also known as LAL-test—is an alternative in vitro endotoxin assay, accepted by the main regulatory drug agencies (FDA, European Medicines Agency (EMA) or Pharmaceuticals and Medical Devices Agency (PMDA), among others). There are three techniques to perform this test, all of them based on the *Limulus* amebocyte lysate (LAL). In 1964, Levin and Bang first recognized the coagulation of the lysate in the presence of GNB endotoxin [7,8]. However, some complex formulations such as radiopharmaceuticals, biotechnology formulations, etc. cannot be assayed by BET. Despite scientist having proposed several in vitro methods which are faster and more easily automated than the original LAL-tests of Cooper and Mills, the latter still remains the only official in the main pharmacopeias [9,10,11,12,13,14,15,16,17].

FDA and EMA have considered the monocyte activation test (MAT) as a humane alternative method to RPT [18,19]. The assay involves incubating a diluted test sample with a source of human monocytes or human monocytoid cells. Monocytes activated by pyrogens produce cytokines/interleukins that are detected in an immunological assay. Human whole blood, peripheral blood mononuclear cells (PBMC) or monocytic cell lines, are appropriate sources of monocytes and are commercially available [20,21,22,23,24]. As MAT has higher sensitivity and accuracy than RPT, promises to replace it in the near future.

Although some interesting reviews about the RPT and the BET were previously published, to our knowledge, none of them provide an in-depth comparison of the two currently available official methods to evaluate pyrogenicity (RPT and BET). The goal of this review is to highlight the differences and similarities of each assay throughout the USP, European Pharmacopoeia (EP), Japanese Pharmacopoeia (JP) and International Pharmacopoeia (IP), taking into account the current thinking of the regulatory authorities, as well as the suggestions and recommendations of the International Council for Harmonisation of Technical Requirements for Pharmaceuticals for Human Use (ICH).

## 2. In Vivo Pyrogen Assay: Rabbit Pyrogen Test (RPT)

In Table 1, Table 2, Table 3 and Table 4, comparative specifications, procedures, and characteristics have been extracted from the official monographs regarding animals and good laboratory practice (GLP) conditions, temperature recording, acceptance criteria, and judgement [25,26,27,28].

Among the evaluated pharmacopoeias, the most significant differences related to the experimental conditions for the animals involved in the assay are housing temperature (USP and JP the most restrictive), feeding during housing (only the EP demands a diet without antibiotics), and initial rabbit rejection reasons (the IP and the EP are the most restrictive).

For the experimental conditions regarding temperature recording, the most important differences among the selected pharmacopoeias are: the depth of the temperature recorder device, the feeding and the watering. These factors can influence the obtained results significantly.

There are also important differences in the RPT procedure among the main pharmacopoeias. From our point of view, the most important ones are the pre-training, the pre-injection conditioning time and the injecting time.

Regarding the acceptance criteria and judgement, the main differences are the number of rabbits in the extra-group and above all, the acceptance criteria.

It should be noted that the USP and the EP make some remarks about the number of rabbits, the overall treatment of the rabbits, and the replacement of the rabbit pyrogen test by an “in vitro” test. In addition, the USP is the only test to give instructions for pyrogen testing of medical devices, injection assemblies and radioactive pharmaceuticals.

## 3. In Vitro Pyrogen Assay: Bacterial Endotoxins Test (BET)

Bacterial Endotoxins Test is completely harmonized according to the Q4B annex 14 published by the ICH in 2012 [29]. In the IP and USP there are three possible alternatives: The gel-clot technique, which is based on gel formation; the turbidimetric technique, based on the development of turbidity after cleavage of an endogenous substrate; and the chromogenic technique, based on the development of color after cleavage of a synthetic peptide-chromogen complex [30,31]. The JP outlines two detailed assays: the gel-clot techniques, which are based on gel formation by the reaction of the lysate TS with endotoxins and the photometric techniques, based on endotoxin-induced optical changes of the lysate TS. In the EP and related pharmacopoeias (The British Pharmacopoeia (BP), Real Farmacopea Española (RFE), etc.), six methods are described: Method A, or gel-clot method limit test; method B or gel-clot method quantitative test; method C, or turbidimetric kinetic method; method D or chromogenic kinetic method; method E, or chromogenic end-point method; and method F, or turbidimetric end-point method [32,33]. In all the pharmacopoeias, the gel-clot limit test should be used if there are doubts about the results of the other proposed methods.

Regarding the apparatus, reagents, test solutions and determination of maximum valid dilution (MVD), no differences between the four pharmacopoeias can be determined. Authors only find little variations between JP and the other pharmacopoeias in some specific points (Table 5 and Tables S1–S3).

## 4. Conclusions

The RPT is not harmonized between the IP and the ICH pharmacopoeias (JP, USP and EP). There are important differences in the design, procedure, temperature recording, acceptance criteria and judgement. Although the current thinking of the regulatory authorities is the transition from the in vivo test to a validated in vitro test, such as the LAL-test in accordance with 21 CFR 610.9. Nowadays, the only way for some products to demonstrate apyrogenicity during the preclinical phase is the RPT, especially if the risk assessment indicates that non-endotoxin pyrogens may be present. In Europe, the EP has an alternative test to the rabbit test. This is the monocyte activation test, a whole blood assay. Thus, pharmaceutical laboratories should consider these differences in their dossiers.

The harmonized ICH-BET, the most popular quality control endotoxin test, has as expected no significant differences across the published official monographs, and all of them may be considered interchangeable. Nevertheless, the pharmaceutical companies should demonstrate to the regulatory authorities that the selected method is acceptable and suitable for a specific material or formulation.

## Figures and Tables

**Table 1:** Experimental conditions referring to animals involved in the assay.

Experimental	IP	USP	JP	EP
Number	3	3	3	3
Condition	Healthy	Healthy	Healthy	Healthy
Age	Adult	Mature	Mature	Adult
Weight	-	-	≥1.5 kg	≥ 1.5kg
Sex	-	-	-	either
Variety	The same, ideally	-	-	-
**Housing:**				
temperature	uniform (±2 °C)	uniform (20–23 °C) (±3 °C)	constant = 20 °C–27 °C	uniform, appropriate
humidity	uniform	-	-	-
watering	*ad libitum*	-	-	-
feeding	usual food *ad libitum*	-	constant diet	complete, balanced, antibiotics free diet
Environs	Not exciting	Not exciting	Not exciting	Quiet
Individually or in group	Individually	Individually	Individually	Individually
Rejection reasons	▪ Last use, in a pyrogen test, in the last 48 h ▪ T rise in the last test ≥ 0.5 °C ▪ Last use in a pyrogen-positive test in the previous 2 weeks	▪ Last use, in a pyrogen test, in the last 48 h ▪ T rise ≥ 0.6 °C in a pyrogen test in the previous 2 weeks ▪ Last use in a pyrogen-positive test in the previous 2 weeks	▪ Last use, in a pyrogen test, in the last 48 h ▪ Last use in a pyrogen-positive test ▪ Loss of body mass in the previous week	▪ Loss of body mass in the previous week ▪ Last use, in a pyrogen test, in the last 3 days ▪ Last use, in a pyrogen-positive test, in the last 3 weeks ▪ Use, in a pyrogen test, where the rabbits’ temperature mean rise > 1.2 °C

IP: International Pharmacopoeia; USP: United States Pharmacopoeia; JP: Japanese Pharmacopoeia; EP: European Pharmacopoeia.

**Table 2:** Experimental conditions referring to temperature recording in the rabbit pyrogen test (RPT).

Experimental	IP	USP	JP	EP
Test room	Housing area or similar separate room	Separate area designated solely for pyrogen testing	Separate room	Housing area or separate room (at least 18h of previous staying)
Room T	Similar to the housing T	Similar to the housing T	Similar to the housing T	Within 3 °C of the housing T
Instrument	Accurate thermometer or T-recording device	Accurate T-sensing device (clinical thermometer or thermistor probe)	Rectal thermometer or T-measuring apparatus	Thermometer or electrical device
Precision	0.1 °C	± 0.1 °C	≤ ± 0.1 °C	0.1 °C
Time	Sufficient to reach a maximum T	Sufficient to reach a maximum T and < 5 min	-	An electrical device may be left throughout the test.
Site and depth	Rectum, ≈ 6 cm	Rectum, ≥ 7.5 cm	Rectum, 6–9 cm constant	Rectum, ≈ 5 cm constant
Restraint	By a loosely fitting neck stock	With lightly fitting neck stock	By a loosely fitting neck stock	By a loosely fitting neck stock (at least 1h before and throughout the test)
Posture	Natural resting	Natural resting	Natural resting	Normal
Feeding	Not allowed (2h before and during test)	Not allowed	Not allowed (several hours before and during test)	Not allowed (previous overnight and during test)
Watering	Allowed	Allowed, may be restricted	-	Not allowed

T: temperature; IP: International Pharmacopoeia; USP: United States Pharmacopoeia; JP: Japanese Pharmacopoeia; EP: European Pharmacopoeia.

**Table 3:** RPT procedure.

Experimental	IP	USP	JP	EP
Pretraining for rabbits not previously used	Same test omitting the injection, 1–3 days before	Same test omitting the injection, not more than 7 days before; for rabbits never used before	Same test omitting the injection, 1–3 days before	Same test injecting pyrogen-free 9 g/L solution of sodium chloride R, 1–3 days before. Non-use period = 2 weeks
Pre-injection conditioning time	≥ 1 h	-	≥ 48 h	18 h
Control T (CT)	Mean of two T readings (T1, T2) at an interval of 30 min in the 40 min preceding the injection	Taken no more than 30 min prior to the injection	Mean of two T readings (T1, T2) at an interval of 30 min in the 40 min preceding the injection	Mean of two T readings (T1, T2) at an interval of 30 min in the 40 min preceding the injection
Rabbit selection criteria	▪ ΔCT among rabbits ≤ 1.0 °C ▪ T1-T2 ≤ mean ± 0.2 °C ▪ 38 °C ≤ CT ≤ 39.8 °C	▪ ΔCT among rabbits ≤ 1.0 °C ▪ CT < 39.8 °C	▪ T1-T2 ≤ ± 0.2 °C ▪ CT ≤ 39.8 °C	▪ ΔT ≤ 0.6 °C in the pretraining ▪ T1-T2 ≤ ± 0.2 °C ▪ 38 °C ≤ CT ≤ 39.8 °C ▪ ΔCT among rabbits ≤ 1.0 °C
Syringe, needle and glassware	Free of pyrogens by any suitable method (250 °C, 30 min)	Free of pyrogens by any suitable method (250 °C, 30 min)	Free of pyrogens	Thorough wash and heating in a hot-air oven (250 °C, 30 min or 200 °C, 1h)
Test material	Solution of the substance being examined	Either the product or the product treated as directed in the monograph	Solution of the substance being examined. When hypotonic, may be made isotonic.	Sterile solution of the substance being examined
Tested product amount	As specified in the monograph	As prescribed in the monograph	-	As prescribed in the monograph
Volume injected	10 mL/kg (or as specified in the monograph)	10 mL/kg (or as specified in the monograph)	10 mL/kg (or as specified in the monograph)	0.5 mL/kg–10 mL/kg
Test solution T	≈ 38 °C	37 °C ± 2 °C	37 °C ± 2 °C	≈ 38 °C
Injection site	Marginal vein of the ear	Ear vein	Marginal vein of the ear	Marginal vein of the ear
Injecting time	≤ 4 min (or as specified in the monograph)	≤ 10 min	≤ 10 min	≤ 4 min (or as specified in the monograph)
Measurement period, after injection	3 h	3 h	3 h	3 h
Measurement frequency	Continuously or every 30 min	Every 30 min between 1 and 3 h subsequent to the injection	≤ 30 min	≤ 30 min (starting at least 90 min before the injection)
Rabbit T rise (TR) = response	▪ TR = T_max_ − CT ▪ TR = 0 when T_max_ < CT (T_max_ = maximum T recorded after injection/rabbit)	▪ TR = T − CT ▪ TR = 0 when T < CT (T = any T recorded after injection/rabbit)	▪ TR = T_max_ − CT ▪ TR = 0 when T_max_ < CT (T_max_ = maximum T recorded after injection/rabbit)	▪ TR = T_max_ − CT ▪ TR = 0 when T_max_ < CT (T_max_ = maximum T recorded after injection/rabbit)

T: temperature; IP: International Pharmacopoeia; USP: United States Pharmacopoeia; JP: Japanese Pharmacopoeia; EP: European Pharmacopoeia

**Table 4:** RPT acceptance criteria and judgement.

Experimental	IP	USP	JP	EP
Number of rabbits extra-groups	1	1	Up to 2	Up to 3
Number of rabbits per extra-group	5	5	3	3
Case 1 and judgment	▪ No individual TR ≥ 0.6 °C **and** Σ TR ≤ 1.4 °C ▪ Absence of pyrogens	▪ No individual TR ≥ 0.5 °C ▪ Absence of pyrogens	▪ Σ TR ≤ 1.3 °C ▪ Pyrogen-negative	▪ Σ TR (n = 3, 6, 9 or 12) ≤ 1.15, 2.80, 4.45 or 6.60 (°C) respectively ▪ Product passes
Case 2 and judgment	▪ 1 or 2 individual TR ≥ 0.6 °C **or** Σ TR (n = 3) > 1.4 °C ▪ Test 5 other rabbits	▪ Any individual TR ≥ 0.5 °C ▪ Test 5 other rabbits	▪ Σ TR > 2.5 °C ▪ Pyrogen-positive	▪ Σ TR (n = 3, 6, 9 or 12) > 2.65, 4.30, 5.95 or 6.60 (°C) respectively ▪ Product fails
Case 3 and judgment	▪ Not more than 3 of the TR (n = 8) ≥ 0.6 °C **and** Σ TR (n = 8) ≤ 3.7 °C ▪ Absence of pyrogens	▪ Not more than 3 of the TR (n = 8) ≥ 0.5 °C **and** Σ TR (n = 8) ≤ 3.3 °C ▪ Absence of pyrogens	▪ 1.3 °C < Σ TR < 2.5 °C ▪ Test 3 other rabbits	▪ Σ TR does not meet neither case 1 nor case 2 ▪ Test 3 other rabbits up to 12
Case 4 and judgment	-	-	▪ Σ TR (n = 6) ≤ 3.0 °C ▪ Pyrogen-negative	-
Case 5 and judgment	-	-	▪ Σ TR (n = 6) > 4.2 °C ▪ Pyrogen-positive	-
Case 6 and judgment	-	-	▪ 3.0 °C < Σ TR (n = 6) < 4.2 °C ▪ Test 3 other rabbits	-
Case 7 and judgment	-	-	▪ Σ TR (n = 9) ≤ 5.0 °C ▪ Pyrogen-negative	-
Case 8 and judgment	-	-	▪ Σ TR (n = 9) > 5.0 °C ▪ Pyrogen-positive	-

TR. Rabbit temperature rise; IP: International Pharmacopoeia; USP: United States Pharmacopoeia; JP: Japanese Pharmacopoeia; EP: European Pharmacopoeia.

**Table 5:** Differences in harmonized bacterial endotoxins test (BET) between JP and IP, USP and EP.

Experimental	IP-USP-EP	JP
Gel-clot techniques: valid test conditions	The lowest concentration of the standard solutions shows a (-) result	When 0.25λ of the standard solution shows a (-) result
Photometric quantitative techniques: requirements	▪ Sol. C comply assurance of criteria ▪ endotoxin recovery: 50–200% ▪ Sol. D: ≤ blank value of the lysate employed or < endotoxin detection limit	▪ |r| of sol. C: ≥ 0.980 ▪ endotoxin recovery: 50–200% ▪ Sol. D: ≤ blank value of the lysate employed or < endotoxin detection limit

IP: International Pharmacopoeia; USP: United States Pharmacopoeia; JP: Japanese Pharmacopoeia; EP: European Pharmacopoeia.

## Supplementary Materials

The following is available online at http://www.mdpi.com/2072-6651/10/8/331/s1, Table S1: Comparative analysis of the BET conditions between IP, USP, JP, EP and also BP and RFE, Table S2: Comparative analysis of the Gel-clot techniques between IP, USP, JP, EP and also BP and RFE, Table S3: Comparative analysis of the Photometric quantitative techniques between IP, USP, JP, EP and also BP and RFE.
