# Supplementary material for: Endotoxins from a Pharmacopoeial Point of View
Source: Toxins (Basel). 2018 Aug 16;10(8):331. doi: 10.3390/toxins10080331 (PMC6115822; doi:10.3390/toxins10080331)
Supplement: Supplementary file 1 [file toxins-10-00331-s001.pdf]

Supplementary Materials: Endotoxins from a Pharmacopoeial Point of View

Elvira Franco, Verónica Garcia-Recio, Pilar Jiménez, Manuel Garrosa, Tomás Girbés, Manuel Cordoba-Diaz and Damián Cordoba-Diaz

Table S1. Comparative analysis of the BET conditions between IP, USP, JP, EP and also BP and RFE

**Conditions**		**IP**	**USP**	**JP**	**EP**	**BP**	**RFE**
**Apparatus**		Hot-air oven<br>(30 min at 250 °C)					
**Reagents**		▪ Amoebocyte lysate<br>▪ Water for bacterial endotoxins test (BET)<br>▪ Lysate solution	▪ Amoebocyte lysate<br>▪ Water for bacterial endotoxins test (BET)<br>▪ Lysate solution		▪ Amoebocyte lysate<br>▪ Water for bacterial endotoxins test (BET)<br>▪ Lysate solution	▪ Amoebocyte lysate<br>▪ Water for bacterial endotoxins test (BET)<br>▪ Lysate solution	▪ Amoebocyte lysate<br>▪ Water for bacterial endotoxins test (BET)<br>▪ Lysate solution
**Test solutions**	**Standard Endotoxin Stock Solution**	From the WHO International Standard for endotoxin or an endotoxin reference standard that has been calibrated against the WHO International Standard for endotoxin.	From a USP Endotoxin Reference Standard that has been calibrated to the current WHO International Standard for Endotoxin.	From Japanese Pharmacopoeia Reference Standard Endotoxin that has been calibrated to the current WHO International Standard for Endotoxin, using water for BET.	From an endotoxin reference standard that has been calibrated against the International Standard, for example endotoxin standard BRP.	From an endotoxin reference standard that has been calibrated against the International Standard, for example endotoxin standard BRP.	From an endotoxin reference standard that has been calibrated against the International Standard, for example endotoxin standard BRP.
	**Standard Endotoxin Solution**	Serial dilutions of the standard endotoxin stock solution using water for BET					
	**Sample Solutions**	▪ Dissolve or dilute the pharmaceutical substance or the finished preparation using water BET.<br>▪ Adjust pH (6.0-8.0)	▪ Dissolve or dilute drugs using water for BET<br>▪ Adjust pH (6.0-8.0)	▪ Dissolve or dilute drugs using water for BET<br>▪ Adjust pH (6.0-8.0)	▪ Dissolve or dilute active substances or medicinal products using water for BET	▪ Dissolve or dilute active substances or medicinal products using water for BET<br>▪ Adjust pH (6.0-8.0)	▪ Dissolve or dilute active substances or medicinal products using water for BET<br>▪ Adjust pH (6.0-8.0)

					▪ Adjust pH (6.0-8.0)		
**Determination of Maximum Valid Dilution (MVD)**		$$MVD = \frac{endotoxin\ limit\ x\ concentration\ of\ test\ solution}{\lambda}$$ ▪ ***Endotoxin limit:*** for parenteral preparations, defined on the basis of dose = $\frac{K}{M}$ K: threshold pyrogenic dose of endotoxin per kilogram of body mass M: maximum recommended bolus dose of product per kilogram of body mass ▪ ***Concentration of test solution:*** – mg/mL if the endotoxin limit is specified by mass (IU/mg), – Units/mL if the endotoxin limit is specified by unit of biological activity (IU/Unit), – ml/mL if the endotoxin limit is specified by volume (IU/mL). ▪ ***λ:*** the labelled lysate sensitivity in the gel-clot technique (IU/mL) or the lowest concentration used in the standard curve of the turbidimetric or chromogenic techniques.					

Table S2: Comparative analysis of the Gel-clot techniques between IP, USP, JP, EP and also BP and RFE

**Gel-clot techniques**		**IP**	**USP**	**JP**	**EP**	**BP**	**RFE**
Limit test		Method A			Method A	Method A	Method A
Quantitative test					Method B	Method B	Method B
**Preparatory testing**							
**Confirmation of the labelled lysate sensitivity (λ)**							
Replicates		4	4	-	4	4	4
Standard solutions (concentrations equivalent)		2λ, λ, 0.5λ, 0.25λ	2λ, λ, 0.5λ, 0.25λ	2λ, λ, 0.5λ, 0.25λ	2λ, λ, 0.5λ, 0.25λ	2λ, λ, 0.5λ, 0.25λ	2λ, λ, 0.5λ, 0.25λ
Procedure		Mix 1 volume Lysate solution + 1 volume standard solution	Mix 1 volume Lysate TS + 1 volume standard solution	Mix 1 volume Lysate TS + 1 volume standard solution	Mix 1 volume Lysate solution + 1 volume standard solution	Mix 1 volume Lysate solution + 1 volume standard solution	Mix 1 volume Lysate solution + 1 volume standard solution
Conditions:<br>Temperature<br>Time		37 ± 19C<br>60 ± 2 minutes	37 ± 19C<br>60 ± 2 minutes	37 ± 19C<br>60 ± 2 minutes	37 ± 19C<br>60 ± 2 minutes	37 ± 19C<br>60 ± 2 minutes	37 ± 19C<br>60 ± 2 minutes
Results (Invert each tube 180°)	Gel	+	+	+	+	+	+
	No gel	-	-	-	-	-	-
	Valid test	The lowest concentration of the standard solutions shows a (-) result	The lowest concentration of the standard solutions shows a (-) result	When 0.25λ of the standard solution shows a (-) result	The lowest concentration of the standard solutions shows a (-) result	The lowest concentration of the standard solutions shows a (-) result	The lowest concentration of the standard solutions shows a (-) result
	Endpoint	The lowest concentration that clots the lysate	The smallest concentration that clots the lysate	The last (+) test	The lowest concentration that clots the lysate	The lowest concentration that clots the lysate	The lowest concentration that clots the lysate
Geometric mean end-point concentration		**Geometric mean end-point concentration = antilog** $\frac{\Sigma e}{f}$					

	**∑e=** The sum of the log endpoint concentrations of the dilution series used<br><br>**f:** The number of replicates<br><br>If the geometric mean endpoint concentration is $>0.5\lambda$ and $<2\lambda$, the labeled sensitivity is confirmed, and is used in tests performed with this lysate.					
**Test for interfering factors**	Prepare the solutions (A-D) and perform the inhibition/ enhancement test on the sample solutions at a dilution less than the MVD not containing any detectable endotoxins	Prepare the solutions (A-D) and perform the inhibition/ enhancement test on the sample solutions at a dilution less than the MVD not containing any detectable endotoxins	Prepare the solutions A, B, C and D	Prepare the solutions A, B, C and D and use the test solutions at a dilution less than the MVD not containing any detectable endotoxins	Prepare the solutions A, B, C and D and use the test solutions at a dilution less than the MVD not containing any detectable endotoxins	Prepare the solutions A, B, C and D and use the test solutions at a dilution less than the MVD not containing any detectable endotoxins
<u>Solution A:</u><br>▪ Endotoxin concentration / Solution to which endotoxin is added<br>▪ Diluent<br>▪ Dilution factor<br>▪ Endotoxin concentration<br>▪ Replicates	None / test solution<br>-<br>-<br>-<br>4	None / sample solution<br>-<br>-<br>-<br>4	0 / sample solution<br>-<br>-<br>-<br>4	None / test solution<br>-<br>-<br>-<br>4	None / test solution<br>-<br>-<br>-<br>4	None / test solution<br>-<br>-<br>-<br>4
<u>Solution B:</u><br>▪ Endotoxin concentration / Solution to which endotoxin is added<br>▪ Diluent	2λ / test solution<br>Test solution	2λ / sample solution<br>Sample solution	2λ / sample solution<br>Sample solution	2λ / test solution<br>Test solution	2λ / test solution<br>Test solution	2λ / test solution<br>Test solution

▪ Dilution factors<br>▪ Endotoxin concentrations<br>▪ Replicates	1-2-4-8<br>2λ-1λ-0.5λ-0.25λ<br>4	1-2-4-8<br>2λ-1λ-0.5λ-0.25λ<br>4	1-2-4-8<br>2λ-1λ-0.5λ-0.25λ<br>4	1-2-4-8<br>2λ-1λ-0.5λ-0.25λ<br>4	1-2-4-8<br>2λ-1λ-0.5λ-0.25λ<br>4	1-2-4-8<br>2λ-1λ-0,5λ-0,25λ<br>4
<u>Solution C:</u><br>▪ Endotoxin concentration / Solution to which endotoxin is added<br>▪ Diluent<br>▪ Dilution factors<br>▪ Endotoxin concentrations<br>▪ Replicates	 2λ / water for BET<br>Water for BET<br>1-2-4-8<br>2λ-1λ-0.5λ-0.25λ<br>2	 2λ / water for BET<br>Water for BET<br>1-2-4-8<br>2λ-1λ-0.5λ-0.25λ<br>2	 2λ / water for BET<br>Water for BET<br>1-2-4-8<br>2λ-1λ-0.5λ-0.25λ<br>2	 2λ / water for BET<br>Water for BET<br>1-2-4-8<br>2λ-1λ-0.5λ-0.25λ<br>2	 2λ / water for BET<br>Water for BET<br>1-2-4-8<br>2λ-1λ-0.5λ-0.25λ<br>2	 2λ / water for BET<br>Water for BET<br>1-2-4-8<br>2λ-1λ-0,5λ-0,25λ<br>2
<u>Solution D:</u><br>▪ Endotoxin concentration / Solution to which endotoxin is added<br>▪ Diluent<br>▪ Dilution factor<br>▪ Endotoxin concentration<br>▪ Replicates	 None / water for BET<br>-<br>-<br>-<br>2	 None / water for BET<br>-<br>-<br>-<br>2	 0 / water for BET<br>-<br>-<br>-<br>2	 None / water for BET<br>-<br>-<br>-<br>2	 None /water for BET<br>-<br>-<br>-<br>2	 None / water for BET<br>-<br>-<br>-<br>2

Results	▪ Test valid when A and D show no reaction and C confirms the labelled sensitivity<br>▪ Sol. B: if the sensitivity of the lysate is >0.5λ and <2λ the sample solution does not contain interfering factors<br>▪ If the sample under test does not comply with the test at a dilution less than the MVD, repeat the test using a greater di- lution, not exceeding the MVD.	▪ Test valid when A and D show no reaction and C confirms the labelled sensitivity<br>▪ Sol. B: if the sensitivity of the lysate is >0.5λ and <2λ the sample solution does not contain interfering factors<br>▪ If the sample under test does not comply with the test at a dilution less than the MVD, repeat the test using a greater di- lution, not exceeding the MVD.	▪ Test valid when A and D show no reaction and C confirms the labelled sensitivity<br>▪ Sol. B: if the sensitivity of the lysate is >0.5λ and <2λ the sample solution does not contain interfering factors<br>▪ If the sample under test does not comply with the test at a dilution less than the MVD, repeat the test using a greater di- lution, not exceeding the MVD.	▪ Test valid when both replicates of B and C are (+) and those of D are (-).<br>▪ When a (-) result is found for both replicates of A, the preparation complies with the test. When a (+) result is found the preparation does not comply with the test.<br>▪ When a (+) result is found for one replicate of A and a (-) result is found for the other, repeat the test. In the repeat test, the preparation complies with the test if a (-) result is found for both replicates of solution A.	▪ Test valid when A and D show no reaction and C confirms the labelled sensitivity<br>▪ Sol. B: if the sensitivity of the lysate is >0.5λ and <2λ the sample solution does not contain interfering factors<br>▪ If the sample under test does not comply with the test at a dilution less than the MVD, repeat the test using a greater dilution, not exceeding the MVD.	▪ Test valid when A and D show no reaction and C confirms the labelled sensitivity<br>▪ Sol. B: if the sensitivity of the lysate is >0.5λ and <2λ the sample solution does not contain interfering factors<br>▪ If the sample under test does not comply with the test at a dilution less than the MVD, repeat the test using a greater dilution, not exceeding the MVD.
**Limit test**	**IP**	**USP**	**JP**	**EP**	**BP**	**RFE**
**Procedure**	Prepare solutions A, B, C and D.<br>Follow the procedures:<br>▪ Confirmation of the labelled lysate sensitivity<br>▪ Test for interfering factors	Prepare solutions A, B, C and D.<br>Follow the procedures:<br>▪ Confirmation of the labelled lysate sensitivity<br>▪ Test for interfering factors	Prepare solutions A, B, C and D.<br>Follow the procedures:<br>▪ Confirmation of the labelled lysate sensitivity<br>▪ Test for interfering factors	Prepare solutions A, B, C and D.<br>Follow the procedures:<br>▪ Confirmation of the labelled lysate sensitivity<br>▪ Test for interfering factors	Prepare solutions A, B, C and D.<br>Follow the procedures:<br>▪ Confirmation of the labelled lysate sensitivity<br>▪ Test for interfering factors	Prepare solutions A, B, C and D.<br>Follow the procedures:<br>▪ Confirmation of the labelled lysate sensitivity<br>▪ Test for interfering factors

Solution A:<br>Endotoxin concentration/ Solution to which endotoxin is added<br>Replicates	None / Diluted test solution<br>2	None / Diluted sample solution<br>2	0 / Sample solution<br>2	None / Diluted test solution<br>2	None / Diluted test solution<br>2	None / Diluted test solution<br>2
Solution B:<br>Endotoxin concentration/ Solution to which endotoxin is added<br>Replicates	2λ / Diluted test solution<br>2	2λ / Diluted sample solution<br>2	2λ / sample solution<br>2	2λ / Diluted test solution<br>2	2λ / Diluted test solution<br>2	2λ / Diluted test solution<br>2
Solution C:<br>Endotoxin concentration/ Solution to which endotoxin is added<br>Replicates	2λ / water for BET<br>2	2λ / water for BET<br>2	2λ / water for BET<br>2	2λ / water for BET<br>2	2λ / water for BET<br>2	2λ / water for BET<br>2
Solution D:<br>Endotoxin concentration/ Solution to which endotoxin is added<br>Replicates	None / water for BET<br>2	None / water for BET<br>2	0 / water for BET<br>2	None / water for BET<br>2	None / water for BET<br>2	None / water for BET<br>2
**Interpretation**	Test valid:<br>▪ both replicates of B and C are (+) and those of D are (-).<br>▪ (-) result for both replicates of sol. A	Test valid:<br>▪ both replicates of B and C are (+) and those of D are (-).<br>▪ (-) result for both replicates of sol. A	Test valid:<br>▪ both replicates of B and C are (+) and those of D are (-).<br>▪ (-) result for both replicates of sol. A.<br>A (+) result for one replicate	Test valid:<br>▪ both replicates of B and C are (+) and those of D are (-).<br>▪ (-) result for both replicates of sol. A	Test valid:<br>▪ both replicates of B and C are (+) and those of D are (-).<br>▪ (-) result for both replicates of sol. A	Test valid:<br>▪ both replicates of B and C are (+) and those of D are (-).<br>▪ (-) result for both replicates of sol. A

	A (+) result for one replicate of A and a (-) result for the other, repeat the test.	A (+) result for one replicate of A and a (-) result for the other, repeat the test.	of sol. A and a (-) result for the other, repeat the test.	A (+) result for one replicate of A and a (-) result for the other, repeat the test.	A (+) result for one replicate of A and a (-) result for the other, repeat the test.	A (+) result for one replicate of A and a (-) result for the other, repeat the test.
**Quantitative test**	**IP**	**USP**	**JP**	**EP**	**BP**	**RFE**
**Procedure**	Prepare solutions A, B, C and D.<br>Test these solutions according to Confirmation of the labelled lysate sensitivity.	Prepare solutions A, B, C and D.<br>Test these solutions following the test Confirmation of the labelled lysate sensitivity.	Prepare solutions A, B, C and D.<br>When preparing solutions A and B, use sample solutions complying with the test for interfering factors.<br>Concerning the test conditions, follow the procedure described in the confirmation of the labelled lysate sensitivity.	Prepare solutions A, B, C and D.<br>Test these solutions according to Confirmation of the labelled lysate sensitivity.	Prepare solutions A, B, C and D.<br>Test these solutions according to Confirmation of the labelled lysate sensitivity.	Prepare solutions A, B, C and D.<br>Test these solutions according to Confirmation of the labelled lysate sensitivity.
<u>Solution A:</u><br>▪ Endotoxin concentration / Solution to which endotoxin is added<br>▪ Diluent<br>▪ Dilution factor<br>▪ Endotoxin concentration<br>▪ Replicates	None / sample solution<br>Water for BET<br>1-2-4-8<br>-<br>2	None / sample solution<br>Water for BET<br>1-2-4-8<br>-<br>2	0 / sample solution<br>Water for BET<br>1-2-4-8<br>-<br>2	None / test solution<br>Water for BET<br>1-2-4-8<br>-<br>2	None / test solution<br>Water for BET<br>1-2-4-8<br>-<br>2	None / test solution<br>Water for BET<br>1-2-4-8<br>-<br>2

<u>Solution B:</u><br>▪ Endotoxin concentration / Solution to which endotoxin is added<br>▪ Diluent<br>▪ Dilution factors<br>▪ Endotoxin concentrations<br>▪ Replicates	2λ / sample solution<br>-<br>1<br>2λ<br>2	2λ / sample solution<br>-<br>1<br>2λ<br>2	2λ / sample solution<br>-<br>1<br>2λ<br>2	2λ / test solution<br>-<br>1<br>2λ<br>2	2λ / test solution<br>-<br>1<br>2λ<br>2	2λ / test solution<br>-<br>1<br>2λ<br>2
<u>Solution C:</u><br>▪ Endotoxin concentration / Solution to which endotoxin is added<br>▪ Diluent<br>▪ Dilution factors<br>▪ Endotoxin concentrations<br>▪ Replicates	2λ / water for BET<br>Water for BET<br>1-2-4-8<br>2λ-1λ-0.5λ-0.25λ<br>2	2λ / water for BET<br>Water for BET<br>1-2-4-8<br>2λ-1λ-0.5λ-0.25λ<br>2	2λ / water for BET<br>Water for BET<br>1-2-4-8<br>2λ-1λ-0.5λ-0.25λ<br>2	2λ / water for BET<br>Water for BET<br>1-2-4-8<br>2λ-1λ-0.5λ-0.25λ<br>2	2λ / water for BET<br>Water for BET<br>1-2-4-8<br>2λ-1λ-0.5λ-0.25λ<br>2	2λ / water for BET<br>Water for BET<br>1-2-4-8<br>2λ-1λ-0.5λ-0.25λ<br>2
<u>Solution D:</u><br>▪ Endotoxin concentration / Solution to which endotoxin is added<br>▪ Diluent<br>▪ Dilution factor	None / water for BET<br>-<br>-<br>-	None / water for BET<br>-<br>-<br>-	0 / water for BET<br>-<br>-<br>-	None /water for BET<br>-<br>-<br>-	None /water for BET<br>-<br>-<br>-	None /water for BET<br>-<br>-<br>-

▪ Endotoxin concentration<br>▪ Replicates	2	2	2	2	2	2
**Calculation and interpretation**	Test is valid if:<br>▪ Both replicates of sol. D are (-)<br>▪ Both replicates of sol. B are (+)<br>▪ Geometric mean end-point concentration of sol. C is in the range of 0.5λ to 2λ	Test is valid if:<br>▪ Both replicates of sol. D are (-)<br>▪ Both replicates of sol. B are (+)<br>▪ Geometric mean end-point concentration of sol. C is in the range of 0.5λ to 2λ	Test is valid if:<br>▪ Both replicates of sol. D are (-)<br>▪ Both replicates of sol. B are (+)<br>▪ Geometric mean end-point concentration of sol. C is in the range of 0.5λ to 2λ	Test is valid if:<br>▪ Both replicates of sol. D are (-)<br>▪ Both replicates of sol. B are (+)<br>▪ Geometric mean end-point concentration of sol. C is in the range of 0.5λ to 2λ	Test is valid if:<br>▪ Both replicates of sol. D are (-)<br>▪ Both replicates of sol. B are (+)<br>▪ Geometric mean end-point concentration of sol. C is in the range of 0.5λ to 2λ	Test is valid if:<br>▪ Both replicates of sol. D are (-)<br>▪ Both replicates of sol. B are (+)<br>▪ Geometric mean end-point concentration of sol. C is in the range of 0.5λ to 2λ
Endotoxin concentration of sol. A:	Calculate the end-point concentration for each replicate by multiplying each end-point dilution factor by λ.	Calculate the end-point concentration for each replicate by multiplying each end-point dilution factor by λ.	The endpoint is defined as the maximum dilution showing the last positive test in the dilution series of solution A	Calculate the end-point concentration for each replicate by multiplying each end-point dilution factor by λ.	Calculate the end-point concentration for each replicate by multiplying each end-point dilution factor by λ.	Calculate the end-point concentration for each replicate by multiplying each end-point dilution factor by λ.
Endotoxin concentration in the sample solution	It is the end-point concentration of the replicates. If the test is conducted with a diluted sample solution, calculate the concentration of endotoxin in the original sample solution by multiplying by the dilution	It is the end-point concentration of the replicates. If the test is conducted with a diluted sample solution, calculate the concentration of endotoxin in the original sample solution by multiplying by the dilution	It is calculated by multiplying the endpoint dilution factor by λ.	It is the end-point concentration of the replicates. If the test is conducted with a diluted sample solution, calculate the concentration of endotoxin in the original sample solution by multiplying by the	It is the end-point concentration of the replicates. If the test is conducted with a diluted sample solution, calculate the concentration of endotoxin in the original sample solution by multiplying by the	It is the end-point concentration of the replicates. If the test is conducted with a diluted sample solution, calculate the concentration of endotoxin in the original sample solution by multiplying by the

	factor.	factor.		dilution factor.	dilution factor.	dilution factor.
If none of dilutions is (+)	Report the endotoxin concentration as less than λ	Report the endotoxin concentration as less than λ	Report the endotoxin concentration as less than $\lambda\times$ the lowest dilution factor of the sample solution	Report the endotoxin concentration as less than λ	Report the endotoxin concentration as less than λ	Report the endotoxin concentration as less than λ
If all dilutions are (+)	Reported as equal to or greater than the largest dilution factor multiplied by λ	Reported as equal to or greater than the largest dilution factor multiplied by λ	reported as equal to or greater than the greatest dilution factor of solution A multiplied by λ.	Reported as equal to or greater than the largest dilution factor multiplied by λ	Reported as equal to or greater than the largest dilution factor multiplied by λ	Reported as equal to or greater than the largest dilution factor multiplied by λ
	The preparation under test meets the requirements of the test if the concentration of endotoxin in both replicates is less than that specified in the individual monograph.	The preparation under test meets the requirements of the test if the concentration of endotoxin in both replicates is less than that specified in the individual monograph.	The sample com- plies with the Bacterial Endotoxins Test if the endotoxin concentration of the sample in both replicates meets the requirement for the endotoxin limit specified in the individual monograph.	The preparation under test meets the requirements of the test if the concentration of endotoxin in both replicates is less than that specified in the individual monograph.	The preparation under test meets the requirements of the test if the concentration of endotoxin in both replicates is less than that specified in the individual monograph.	The preparation under test meets the requirements of the test if the concentration of endotoxin in both replicates is less than that specified in the individual monograph.

Table S3: Comparative analysis of the Photometric quantitative techniques between IP, USP, JP, EP and also BP and RFE

**Photometric quantitative techniques**		**IP**	**USP**	**JP**	**EP**	**BP**	**RFE**
Turbidimetric	End-point	Method B			Method F	Method F	Method F
	Kinetic				Method C	Method C	Method C
Chromogenic	End-point	Method C			Method E	Method E	Method E
	Kinetic				Method D	Method D	Method D
Temperature		37 ± 1 °C					

**Criteria for the standard curve**	**IP**	**USP**	**JP**	**EP**	**BP**	**RFE**
Endotoxin concentrations within the range	3	3	3	3	3	3
Replicates of each standard endotoxin solution	3	3	3	3	3	3
| r|	≥ 0,980	≥ 0,980	≥ 0,980	≥ 0,980	≥ 0,980	≥ 0,980
**Test for interfering factors**	Select an endotoxin concentration at or near the middle of the endotoxin standard curve			Select an endotoxin concentration at or near the middle of the endotoxin standard curve	Select an endotoxin concentration at or near the middle of the endotoxin standard curve	Select an endotoxin concentration at or near the middle of the endotoxin standard curve
Solution A:<br>▪ Endotoxin concentration<br>▪ Solution<br>▪ Replicates	 (-)<br>Sample solution<br>≥ 2	 (-)<br>Sample solution<br>≥ 2	 0<br>Sample solution<br>≥ 2	 (-)<br>Test solution<br>≥ 2	 (-)<br>Test solution<br>≥ 2	 (-)<br>Test solution<br>≥ 2
Solution B:						

▪ Endotoxin concentration<br>▪ Solution<br>▪ Replicates	Middle concentration of the standard curve<br>Sample solution<br>≥ 2	Middle concentration of the standard curve<br>Sample solution<br>≥ 2	Middle concentration of the standard curve<br>Sample solution<br>≥ 2	Middle concentration of the standard curve<br>Test solution<br>≥ 2	Middle concentration of the standard curve<br>Test solution<br>≥ 2	Middle concentration of the standard curve<br>Test solution<br>≥ 2
Solution C:<br>▪ Endotoxin concentration<br>▪ Solution<br>▪ Replicates	≥ 3 concentrations (lowest C is designated λ). Water for BET Each concentration ≥ 2	≥ 3 concentrations (lowest C is designated λ). Water for BET Each concentration ≥ 2	≥ 3 concentrations (lowest C is designated λ). Water for BET Each concentration ≥ 2	≥ 3 concentrations (lowest C is designated λ). Water for BET Each concentration ≥ 2	≥ 3 concentrations (lowest C is designated λ). Water for BET Each concentration ≥ 2	≥ 3 concentrations (lowest C is designated λ). Water for BET Each concentration ≥ 2
Solution D :<br>▪ Endotoxin concentration<br>▪ Solution<br>▪ Replicates	(-)<br>Water for BET<br>≥ 2	(-)<br>Water for BET<br>≥ 2	0<br>Water for BET<br>≥ 2	(-)<br>Water for BET<br>≥ 2	(-)<br>Water for BET<br>≥ 2	(-)<br>Water for BET<br>≥ 2
Result with solution D	▪ ≤ limit of the blank value<br>▪ < endotoxin detection limit	▪ ≤ limit of the blank value<br>▪ < endotoxin detection limit	▪ ≤ limit of the blank value<br>▪ < endotoxin detection limit	▪ ≤ limit of the blank value<br>▪ < endotoxin detection limit	▪ ≤ limit of the blank value<br>▪ < endotoxin detection limit	▪ ≤ limit of the blank value<br>▪ < endotoxin detection limit
$\vert r \vert$ of standard curve generated using solution C	≥ 0,980	≥ 0,980	≥ 0,980	≥ 0,980	≥ 0,980	≥ 0,980
Mean recovery of the added endotoxin	50–200%	50–200%	50–200%	50–200%	50–200%	50–200%

**Procedure**	Follow test for interfering factors					
**Calculation**	Endotoxin concentration of solution A using the standard curve generated by C	Endotoxin concentration of solution A using the standard curve generated by C	Endotoxin concentration of solution A using the standard curve generated by C	Endotoxin concentration of solution A using the standard curve generated by C	Endotoxin concentration of solution A using the standard curve generated by C	Endotoxin concentration of solution A using the standard curve generated by C
Requirements	▪ Sol. C comply assurance of criteria<br>▪ endotoxin recovery: 50–200%<br>▪ Sol. D: ≤ blank value of the lysate employed or < endotoxin detection limit	▪ Sol. C comply assurance of criteria<br>▪ endotoxin recovery: 50–200%<br>▪ Sol. D: ≤ blank value of the lysate employed or < endotoxin detection limit	▪ $\|r\|$ of sol. C: ≥ 0,980<br>▪ endotoxin recovery: 50–200%<br>▪ Sol. D: ≤ blank value of the lysate employed or < endotoxin detection limit	▪ Sol. C comply assurance of criteria<br>▪ endotoxin recovery: 50–200%<br>▪ Sol. D: ≤ blank value of the lysate employed or < endotoxin detection limit	▪ Sol. C comply assurance of criteria<br>▪ endotoxin recovery: 50–200%<br>▪ Sol. D: ≤ blank value of the lysate employed or < endotoxin detection limit	▪ Sol. C comply assurance of criteria<br>▪ endotoxin recovery: 50–200%<br>▪ Sol. D: ≤ blank value of the lysate employed or < endotoxin detection limit
**Interpretation**	Endotoxin concentration of sol. A < endotoxin detection limit	Endotoxin concentration of sol. A < endotoxin detection limit	Endotoxin concentration of sol. A meets the endotoxin detection limit specified in monograph	Endotoxin concentration of sol. A < endotoxin detection limit	Endotoxin concentration of sol. A < endotoxin detection limit	Endotoxin concentration of sol. A < endotoxin detection limit
